# Fas and Fas ligand are highly expressed in lymphocytes from cervical intraepithelial neoplasia and cervical cancer patients: A possible role for immune escaping

**DOI:** 10.22038/IJBMS.2022.61808.13678

**Published:** 2022-03

**Authors:** Carla O. Contreras-Ochoa, Margarita Bahena-Román, Luz Yvette López-Díaz, Alfredo Lagunas-Martínez, Carlos Mojica-Cardoso, Joaquín Manzo-Merino, Kirvis Torres-Poveda, Vicente Madrid-Marina

**Affiliations:** 1 Centro de Investigación sobre Enfermedades Infecciosas, Instituto Nacional de Salud Pública. Av. Universidad 655, Col. Santa María Ahuacatitlán, Cuernavaca, Mor., México; 2 Laboratorio de Patología, Hospital del Niño Morelense. Av. de la Salud 1, Col. Benito Juárez, Emiliano Zapata, Morelos, México; 3 Consejo Nacional de Ciencia y Tecnología (CONACyT)-Instituto Nacional de Cancerología, San Fernando 22, Col. Sección XVI, Tlalpan, Ciudad de México, México; 4 Consejo Nacional de Ciencia y Tecnología (CONACyT)-Instituto Nacional de Salud Pública, Cuernavaca, Morelos, México; # These authors contributed equally to this work

**Keywords:** Cervical intraepithelial – neoplasia, Fas ligand protein, Fas receptor, Gene expression, Uterine cervical neoplasms

## Abstract

**Objective(s):**

Infection with high-risk human papillomavirus is required to develop cervical cancer. Some viruses modulate the Fas/FasL signaling to evade the immune response; the role of these molecules in cervical cancer is not clear. In this study, we measured the expression levels of Fas and FasL mRNA, soluble proteins, and cell surface proteins in peripheral blood mononuclear cells from patients with low- and high-grade squamous intraepithelial lesions and cervical cancer in relation to healthy women, to gain new insights into the role of Fas/FasL in cervical cancer development.

**Materials and Methods:**

Fas/FasL mRNA expression was measured in cervical tissues and peripheral blood mononuclear cells from patients and healthy subjects; serum soluble proteins Fas/FasL were measured by ELISA, and cell-surface protein expression was detected by flow cytometry.

**Results:**

Varying expression levels were found for both molecules. Cervical Fas and FasL mRNA expression was decreased in low- and high-grade lesions, but it was increased in cervical cancer cases. While, systemic Fas mRNA expression increased as malignity progressed; systemic *Fas*L mRNA expression was increased in low- and high-grade lesions, but it was decreased in cancer patients. Soluble FasL levels decreased as lesions progressed, while soluble Fas levels increased. Finally, overexpression of Fas/FasL on the surface of peripheral blood mononuclear cells was found in patients with low-grade lesion with respect to healthy donors.

**Conclusion:**

Fas and FasL act as negative modulators of the immune response, probably by removing specific cytotoxic T lymphocytes against papillomavirus -infected cells and tumor cells.

## Introduction

Cervical cancer (CC) is a malignancy in the female reproductive tract that seriously threatens women’s health and even life. According to the World Health Organization (WHO), there were approximately 570,000 new CC cases and 311,000 deaths worldwide in 2018, with 90% of cases occurring in developing countries (1, 2). The main risk factor for this cancer type is an infection with high-risk human papillomavirus (HR-HPV). However, about 92% of infected women clear HR-HPV, and only 8% of them develop cervical lesions. Thus, a persistent HR-HPV infection is required to develop lesions that progress into CC, and this process involves an impairment of the immune response (3-5). Several factors have been linked to this impaired immunity, including an immunosuppressive microenvironment resulting from secretion of immunosuppressive cytokines by tumor cells, alterations in Toll-like Receptor (TLR)-9 expression, a reduction of E-cadherin, and a reduced expression of the activator CD3-zeta chain in T lymphocytes, recruitment of regulatory T cells, and presence of death molecules that kill specific cytotoxic T lymphocytes by apoptosis activation through Fas/ Fas Ligand (FasL) signaling (3-14). 

Fas is a member of the TNF receptor superfamily; its ligand, FasL, is located on the surface of various cell types; FasL can be cleaved by a metalloproteinase, releasing a soluble ligand (15, 16). The Fas/FasL system regulates peripheral immune homeostasis, the inflammatory response, apoptosis, and non-apoptotic signaling cell death; it is also involved in tumorigenesis (17-19). When FasL binds its receptor on immune cells, it induces apoptosis of malignant or virus-infected cells. Alterations in the expression of Fas and FasL have been found in many cancer types as a mechanism for tumor cells to escape the immune system; thus, Fas/FasL signaling has been explored as a target for anti-cancer therapies (10, 17, 20-25).

To date, the roles of Fas and FasL in CC are not clear; cervical Fas levels have been reported not to change significantly, while FasL overexpression by tumor cells, ranging from 53 to 94%, has been considered as an evasion mechanism through which tumor cells kill cytotoxic T lymphocytes by apoptosis, which has been linked with carcinogenesis development **(**26-28). In addition, variable levels of *Fas *and* FasL* mRNA or protein expression have been found in CC biopsies (29-33). At a systemic level, increased levels of soluble Fas and FasL have been reported in CC patients (34, 35).

Altogether, these findings suggest that expression of Fas/FasL could be a predictor of CC development. To ascertain this, herein we studied mRNA, soluble proteins, and cell surface protein expression of Fas and FasL in peripheral blood mononuclear cells (PBMCs) from low- and high-grade squamous intraepithelial lesion (L-SIL and H-SIL, respectively) and CC patients, and compared them with cells from women with no cervical lesions. 

## Materials and Methods

***Study design and population***

A cross-sectional study was conducted with samples from a biological sample bank built as previously described (36). The Bioethics and Research Committees of the National Institute of Public Health (CI:814) and the INCan (INCan/CC/326/10CB/609) approved the baseline study in which the biological sample bank was built. All participants provided written informed consent for the use of their biological samples in further research studies. Each subject was interviewed for lifestyle, sociodemographic, and reproductive factors. Women with autoimmune diseases, having other sexually transmitted diseases, and who had been given treatment were excluded from the study.

The women included in this study attended the “Centro de Atención para la Salud de la Mujer” (CAPASAM) in Morelos, Mexico, from June 2008 to December 2011, and the “Servicio de Ginecología del Instituto Nacional de Cancerología (INCan)” in Mexico City from September 2010 to December 2011, and from February to May 2015. CC cases (aged 22–86) were diagnosed with either invasive squamous cell carcinoma or invasive adenocarcinoma in the cervix (*n* = 30). Cases of squamous intraepithelial lesions (aged 18–72) were classified as either L-SIL (*n* = 30) or H-SIL (*n* = 30). Patient classification was based on the diagnosis by the corresponding Pathology Department of CAPASAM and INCan. Subjects with no cervical lesions (NCL) and a negative PCR result for HPV (aged 17–68) were included as negative controls (*n* = 30). Cervical epithelial scraping specimens were analyzed to detect HPV DNA. HPV was typified by PCR amplification using consensus primers MY09/11: 5´CGT CCM ARR GCA WAC TGA TC 3´ and 5´ GCM CAG GGW CAT AAY AAT GG 3´ annealing temperature 57 °C, fragment size 450 bp (37), L1C1/L1C2: 5´CGT AAA CGT TTT CCC TAT TTT TTT 3´ and 5´ TAC CCT AAA TAC TAC TCT GTA TTG-3´ annealing temperature 50 °C, fragment size 250 bp (38) and GP5/GP6: 5´ TTT GTT ACT GTG GTA GAT ACT AC-3´ and 5´ GAA AAA TAA ACT GTA AAT CAT ATT C 3´ annealing temperature 40 °C, fragment size 150 bp (39), flanking the HPV L1 gene. PCR products were resolved in 2% agarose and visualized with ethidium bromide staining.

***Characteristics of the biological sample bank***

The biological sample bank used in this study included single-strand complementary DNA (cDNA) samples extracted from cervical epithelial scraping cells (in NCL subjects), from fresh cell biopsies (in SIL and CC cases), and peripheral blood samples collected from the subjects. Peripheral blood samples were collected in serum-separating vacutainer tubes and centrifuged to obtain serum. Total RNA was isolated from PBMCs obtained by density gradient centrifugation using Lymphoprep (Axis-Shield, Oslo, Norway), and from cervical epithelial scrapings and biopsies using the TRIzol reagent (Invitrogen Life Technologies, Carlsbad, CA, USA). cDNA was synthesized according to a previously described protocol (36). Primers for the human housekeeping glyceraldehyde-3-phosphate dehydrogenase gene (GAPDH, 450 pb) Fwd 5´ ACCACAGTCCATGCCATCAC 3´ Rev 5´ CCACCACCCTGTTGCTGTA 3´ (40) was used to verify cDNA integrity. 

***Real-time quantitative PCR for Fas and FasL mRNA***

To assess Fas and FasL expression, a TaqMan real-time quantitative PCR assay was performed using a StepOnePlus™ Real-Time PCR System (Thermo Fisher Scientific, Waltham, MA, USA), following the manufacturer’s directions. Gene expression was normalized against the reference gene *HPRT1*. All primers and the probe were purchased commercially (TaqMan Inventoried Gene Expression Assay FasL: Hs00181225_m1; Fas Hs00531110_m1; and HPRT1, Hs01003267_m1; Applied Biosystems, Waltham, MA, USA). Amplification was carried out in duplicate in a final volume of 10 μl, in 96-well reaction plates, as previously described (36). mRNA expression levels for the genes under study were determined by relative quantification with the comparative Ct method (2^-^^Δ^^Ct^) and plotted as relative units of expression (RUE) for each gene relative to the endogenous *HPRT1* gene and to the control group. All samples were analyzed in duplicate. The values are reported as median ± SE. *P*-values<0.05 are marked with an asterisk.

***sFas and sFasL detection in serum by ELISA***

Serum soluble protein levels were measured with the Quantikine kit (Human Fas Ligand/TNFSF6 and Human sFas/TNFRSF6 immunoassay; R&D Systems, Minneapolis, MN, USA), following the manufacturer’s instructions. All assays were performed in duplicate. Soluble protein levels (pg/ml) are reported as mean ± SD. *P*-values<0.05 are marked with an asterisk.

***Cell-surface antigen staining and flow cytometry***

EDTA-treated blood samples either from healthy subjects or L-SIL patients (*n* = 10 for each group) were analyzed. Whole blood (50 µl) was stained directly for surface antigens with the following monoclonal antibodies**: **anti-human CD95 Alexa Fluor® 488 (Fas, catalog number 305616, Biolegend, San Diego, CA, USA), anti-human CD178 PE (Fas-L, catalog number 306407, Biolegend) and anti-human TBNK BD-Multitest 6-color (CD3 FITC/CD16 PE+CD56 PE/CD45 PerCP-Cy™5.5/CD4 PE-Cy™7/CD19 APC/CD8 APC-Cy™7, catalog number 337166, BD Multitest, Becton Dickinson, NJ, USA**),** following the manufacturer’s instructions. The tubes were gently mixed and incubated at room temperature in the dark for 30 min. Then, the cells were resuspended in 2 ml of FACS lysing solution **(**catalog number 349202, Becton Dickinson, NJ, USA) and then gently mixed and incubated at room temperature in the dark for 10 min. After that, they were centrifuged at 540 × *g* for 5 min. Supernatants were discarded, and the pellets were resuspended in FACS buffer (PBS + 1% bovine serum albumin). Data were acquired in a FACSAria II flow cytometer (Becton Dickinson) and analyzed with the FACS Diva software. Debris and doublets were removed through side (SSC) and forward scatter (FSC) gating (41). Lymphocyte, monocyte/macrophage, and granulocyte populations positive from each marker were determined by acquiring 10,000 events.

***Statistical analysis***

Median values of *Fas* and *FasL* mRNA systemic and cervical expression levels were compared between NCL controls and L-SIL, H-SIL, and CC cases with a Wilcoxon Mann-Whitney test. Mean serum sFas and sFasL concentrations were compared among groups by one-way ANOVA; a Tukey *post-hoc* test was used to compare frequencies among experimental groups. *Fas* and *FasL* mRNA systemic expression was correlated with serum protein concentrations in NCL, L-SIL, H-SIL, and CC cases with a Spearman rank correlation analysis. *P*<0.05 was regarded as statistically significant. All statistical analyses were performed with the software STATA v.14.0 (StataCorp, College Station, TX, USA). All experiments were performed in duplicate.

## Results

***Fas and FasL mRNA expression in cervical lesions and CC***

**Figure 1:**
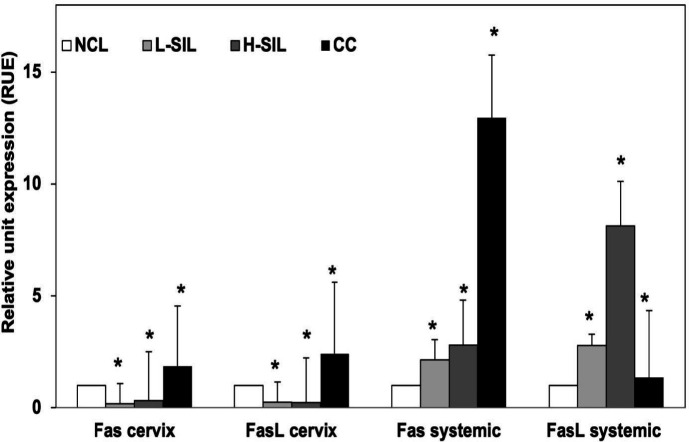
*Fas* and *FasL* mRNA local (cervix) and systemic expression in patients with differing lesion grades and cervical cancer. Medians ± Standard error (*: *P*<0.05). NCL: Non-cervical lesions. L-SIL: Low Squamous Intraepithelial Lesion. H-SIL: High Squamous Intraepithelial Lesion. CC: Cervical cancer

Cervical* Fas* and *FasL* expression levels in NCL, L-SIL, H-SIL, and CC patients, normalized with respect to* HPRT1 *mRNA, are shown in Figure 1. Median cervical expression levels of* Fas* and *FasL* mRNA were significantly different in SIL and CC cases with respect to NCL subjects. The median cervical levels of *Fas* and *FasL *mRNA relative to *HPRT1* were higher in CC patients (1.9 RUE and 2.4 RUE, respectively, *P*=0.00004) than in NCL subjects. Also, median *Fas* and *FasL* expression levels were lower in L-SIL (0.18 RUE and 0.25 RUE, respectively, *P*=0.00001) and H-SIL patients (0.31 RUE and 0.23 RUE, respectively, *P*=0.009) than in NCL subjects. 

***Systemic Fas and FasL mRNA expression***

Statistically significant differences were observed in the median systemic expression levels of *Fas* and *FasL* mRNA between SIL and CC cases and the NCL group (Figure 1). A significant and progressive increase in the systemic levels of *Fas* expression was observed in L-SIL, H-SIL, and CC patients with respect to the NCL group (2.1 RUE, *P*=0.001; 2.8 RUE, *P*=0.00001; and 12.9 RUE, *P*=0.000001, respectively). Interestingly, while increased *FasL* expression levels were observed in L-SIL and H-SIL cases with respect to the NCL group (2.8 RUE, *P*=0.008, and 8.1 RUE, *P* = 000001), they were substantially decreased in CC cases (1.35 RUE, *P*=0.00001). The highest median systemic expression levels of *Fas* and *FasL* mRNA with respect to NCL subjects were found in CC and H-SIL patients, respectively.

***Systemic sFas and sFasL protein levels***

**Figure 2.**
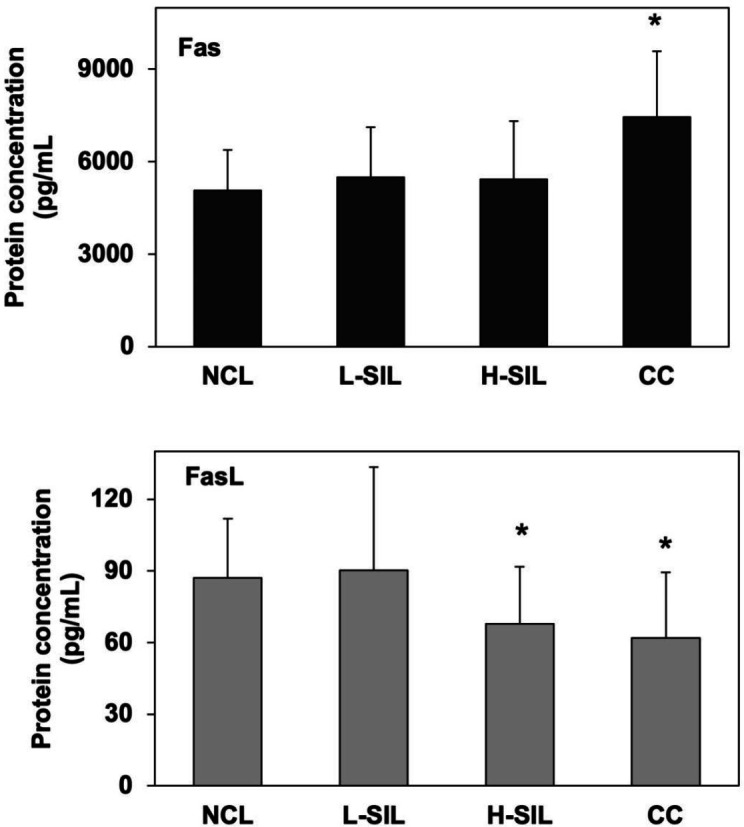
Serum sFas and sFasL levels in patients with differing lesion grades and cervical cancer. Means ± Standard deviation (*: *P*<0.05). Non-cervical lesions. L-SIL: Low Squamous Intraepithelial Lesion. H-SIL: High Squamous Intraepithelial Lesion. CC: Cervical cancer; NCL: Non-cervical lesions

Serum sFas levels increased as the cervix malignancy progressed. Conversely, sFasL serum concentration decreased as the cervix malignancy progressed (Figure 2). Mean serum sFas levels were significantly higher in CC patients (7,449 pg/ml) than in NCL subjects (5,066 pg/ml, *P*=0.001). Statistically significant differences were observed in mean serum sFasL levels between NCL subjects and H-SIL (94.202 pg/ml and 74.152 pg/ml, respectively, *P*=0.01) and CC (68.011 pg/ml, *P*=0.047), except for NCL subjects and L-SIL cases (*P* = 0.61). 

***Correlation between systemic Fas and FasL mRNA expression and serum sFas and sFasL concentration***

To identify a potential correlation between systemic *Fas* and *FasL* mRNA expression and serum sFas and sFasL concentration we carried out a Spearman rank correlation analysis. Only the systemic expression of *Fas* mRNA showed a significant correlation with circulating sFas levels in NCL, L-SIL, H-SIL, and CC patients (*r* = 0.24, *P*=0.03). 

***Fas and FasL protein expression in PBMCs from L-SIL patients***

**Figure 3:**
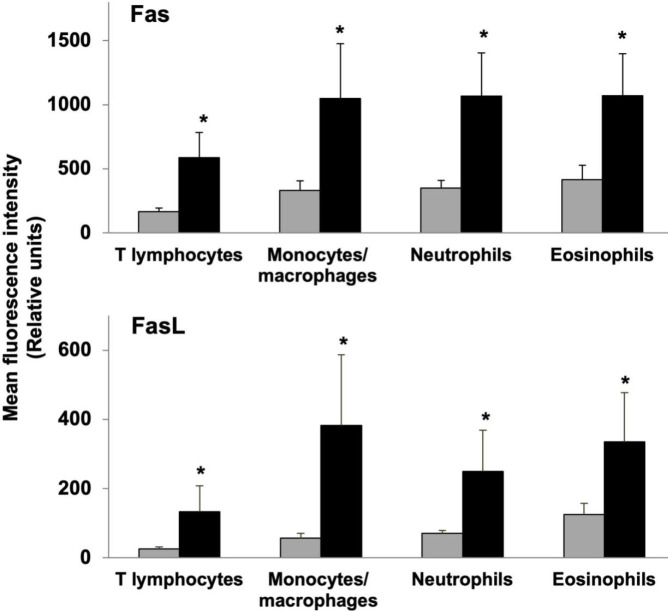
Fas and FasL expression in PBMCs from patients with Low Squamous Intraepithelial Lesion (L-SIL) analyzed by flow cytometry. Means ± Standard deviation (*: *P*<0.05). L-SIL patients (black bars), healthy women (grey bars)

The protein expression of Fas and FasL on the cell surfaces of T lymphocytes, monocytes/macrophages, and granulocytes (neutrophils and eosinophils) was evaluated by flow cytometry. In all cell types analyzed, Fas and FasL expressions were higher in L-SIL patients with respect to healthy donors (Figure 3). A 3.5-fold increase in Fas protein expression was detected in T lymphocytes while a 3-fold increase was shown in monocytes/macrophages and neutrophils, followed by eosinophils (2.6-fold). Similarly, FasL protein was increased on monocytes/macrophages and T lymphocytes (6.8- and 5.1-fold, respectively), followed by neutrophils and eosinophils (3.5- and 2.7-fold, respectively) with respect to healthy individuals.

**Figure 4:**
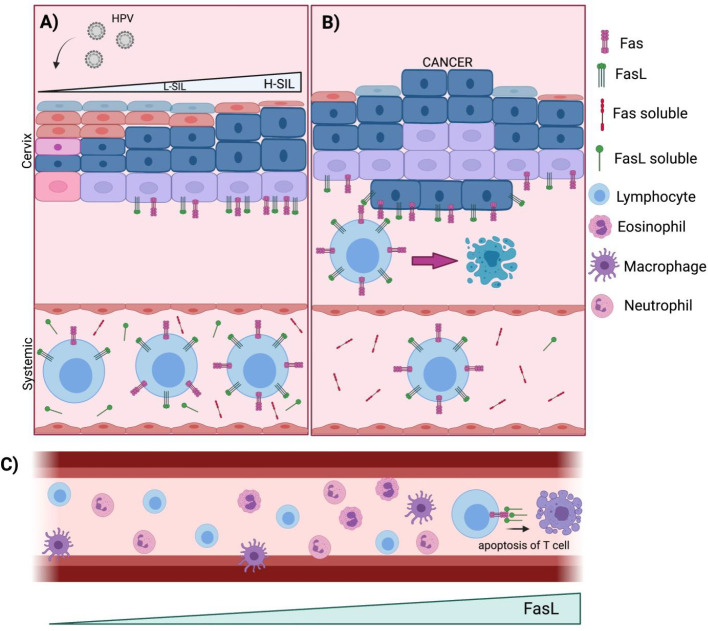
Schematic representation of the role of Fas and FasL in CC progression. The main event in human papillomavirus (HPV)-induced carcinogenesis is the infection of cervical epithelial cells at the basement membrane. Upon infection, an average of 2–3 years are required to develop L-SIL and H-SIL. The immune system plays a key role in HPV-induced carcinogenesis, since more than 90% of high-risk HPV infections regress, as well as most of the low-grade lesions (75%). The long period of time between the viral infection and the development of an invasive disease implies a failed immune response, crucial for cancer progression. A) Upon HPV infection, Fas and FasL are overexpressed at the systemic level, on the surface of immune cells, and in the serum of L-SIL patients, possibly derived from activated lymphocytes in response to HR-HPV infection. B) FasL levels are increased in cervical cancer cells in correspondence with the lesion grade, with the highest concentrations measured in cancer patients. This may lead to release of FasL from cervical cancer cells, which interacts with the Fas receptor on the surface of T lymphocytes, probably inducing apoptosis. C) These events are key for the impairment of immune responses allowing the persistence of HPV and eventually results in cell transformation

## Discussion

Our understanding of the role of Fas and FasL in the development of CC is still limited. In this study, we compared the expression of *Fas *and *FasL *mRNA in cervical tissues and PBMCs from patients with cervical lesions in different stages and CC. We found varying levels of expression for both molecules. The cervical expression of *Fas *and* FasL* mRNA followed a similar trend, being decreased in L-SIL and H-SIL but increased in CC cases. Meanwhile, in PBMCs, Fas and FasL expression increased as malignity progressed, but FasL levels decreased in CC patients. The lower cervical expression of *Fas *and* FasL* mRNA observed at early stages of the disease could indicate that *Fas *and* FasL* mRNA had been partially traduced to the respective protein. This suggests an early activation of the immune response to cervical transformed cells, which is maintained throughout the carcinogenesis process. *Fas *and* FasL* mRNA expression has been poorly studied; in contrast with our results, a down-regulation of *Fas *mRNA expression was observed in tissues from CC patients, while *FasL* mRNA expression levels showed no significant differences with the NCL group (33). Similar results were reported in endometrial carcinoma and ovarian cancer patients (33).

When measuring the serum levels of sFas and sFasL in patients with cervical lesions and healthy controls, sFas levels were increased as cervical malignancy progressed. By contrast, sFasL levels decreased as the malignancy evolved. Correlation analyses between mRNA expression and protein levels for both molecules only showed a relation between systemic *Fas *mRNA expression levels with sFas serum concentrations. As the malignity progressed, a higher *Fas *and *FasL* mRNA expression correlated with higher serum sFas levels, especially in CC patients, while a decreased *FasL* mRNA expression in CC cases correlated with lower serum FasL levels. The decrease of sFasL in patients with CC indicates that this molecule may be bound to Fas on the surface of T lymphocytes, where we found high expression levels of Fas, which may induce T cell apoptosis. In agreement with our results, other authors have reported increased sFas levels in CC (10, 34, 35); however, increased sFasL levels have been observed in CC and other uterine tumors (34), while no differences were found in patients with oral squamous cell carcinoma (42).

While we did not evaluate the local expression of the proteins Fas and FasL in the cervix, other authors have assessed it by immunohistochemistry and reported a different pattern of Fas expression. In most cases, Fas protein expression values were similar in normal tissue and early intraepithelial lesions, but it was slightly increased in H-SIL and/or down-regulated as carcinogenesis progressed. On the other hand, overexpression of FasL was observed as malignancy progressed into CC, accompanied by a down-regulation of apoptosis and the inflammatory response; this resulted in cervical carcinoma cells escaping the immune system, metastasis, a worse prognosis, and a lower patient survival rate (20, 27, 28, 30, 31). Taken together, these findings suggest that deregulation of Fas, overexpression of FasL, and the inverse correlation between FasL overexpression and the presence of CD45^+^ tumor-infiltrating lymphocytes in CC tissues as reported previously (20**), **could indicate that FasL induced apoptosis in local (cervical) lymphocytes to favor the immune evasion of tumor cells.

Our results showed increased levels of both *Fas* mRNA (cervix and systemic) and sFas (serum) in CC. Various alternatively spliced *Fas* mRNAs, which produce sFas lacking the single membrane-spanning domain, have been described. Furthermore, each sFas isoform inhibits FasL-induced apoptosis (43). In CC, it is well known that the HPV-derived E6 and E7 oncoproteins alter several cellular actions through a plethora of interactions with cellular proteins, some of them associated with immune escape and apoptosis. E6 interacts with proteins involved in the extrinsic apoptosis pathway, such as FADD, TNF-R1, and pro-caspase-8 (44). However, whether the E6 oncoprotein induces an increase in *Fas* mRNA expression as an escape mechanism induced by HPV is still unknown.

In this study, both Fas and FasL were found to be overexpressed on the surface of PBMCs from L-SIL patients with respect to healthy, HPV-negative subjects in a similar age range (22–58 years). T lymphocytes and monocytes/macrophages expressed twice as much Fas than FasL. The high amounts of Fas and FasL on the surface of PBMCs were associated with mRNA overexpression for both molecules at the systemic level. On the other hand, only a significant increase was found in the serum level of Fas protein in CC patients. Taken together, our results indicate a clear overexpression in Fas and FasL at a systemic level, both in mRNA and cell surface protein in HPV-positive patients, possibly as a response from activated lymphocytes to the virus. These findings are consistent with those reported previously by Kurmyshkina *et al.* 2017, who showed in H-SIL and CC patients a significant up-regulation in the levels of circulating CD3^+^CD95^+^ (Fas) and CD4^+^CD95^+^ in T lymphocytes, that also were positive for the apoptosis marker annexin-V, with respect to healthy donors, suggesting that CD95-dependent pathway could be associated with CC development (45). Furthermore, an altered proportion of CD4^+^/CD8^+^ T lymphocytes was reported in PBMCs from L-SIL and CC cases, in relation to healthy donors (8); also in women with CC, the CD8^+ ^T cells were predominant compared with CD4^+^ (8); this difference may be explained for the high levels of TGF-**β **in these patients, which induce apoptosis in CD4^+^ lymphocytes, but not in CD8^+ ^T cells (46). Finally, Sun *et al*. (2005) evaluated also by flow cytometry the Fas and FasL protein expression in CD3^+ ^T lymphocytes from healthy donors carrying different SNPs (single nucleotide polymorphisms) for these molecules, finding significant differences in Fas/FasL expression according to the genotype, which could be associated with susceptibility to CC (47). To the best of our knowledge, no evidence has been reported on Fas and FasL expression assessed by flow cytometry on the surface of macrophages or granulocytes from patients with cervical lesions.

In summary, the local decrease in *Fas *and *FasL *mRNA expression suggests that both molecules could be involved in an evasion of the immune system induced by HR-HPV, while the increased levels of Fas and FasL on the surface of circulating PBMCs suggests an activation of the immune system against viral infection. The increase of* Fas* expression in the cervix and PBMCs from CC patients suggests that HPV-infected cells can evade cell death by cytotoxic T lymphocytes through Fas and FasL. Additionally, the increased expression levels of *Fas* and *FasL *at a systemic level and on the surface of T lymphocytes, and the higher cervical levels of FasL in CC patients suggest that HPV-transformed cells could remove tumor-infiltrating lymphocytes (TILs); this is further supported by the lower systemic, but higher cervical *FasL *levels in CC cases, which may target Fas-expressing T lymphocytes, inducing cell death by apoptosis. A schematic representation of the role of Fas and FasL in CC progression is shown in Figure 4.

## Conclusion

The results herein reported strongly support the hypothesis that Fas and FasL play a key role as negative modulators of the local immune response, probably by removing specific cytotoxic T lymphocytes, which in turn could favor the survival and proliferation of tumor cells and CC progression. Further studies are required to ascertain the role of Fas and FasL in different subsets of T lymphocytes from patients with cervical lesions and cancer.

## Authors’ Contributions

COCO, MBR, and LYLD Performed experiments; COCO, MBR, and KTP Designed the experiments and analysed and interpreted data; JMM Helped elaborate the graphical model and critically revised the manuscript; CMC Supported FACS analyses; COCO, MBR, ALM, JMM, KTP, and VMM Wrote the manuscript; VMM Conceived and designed the study and helped with funding acquisition. 

## Conflicts of Interest

The authors declare that they have no conflicts of interest.
